# Impact of the COVID-19 pandemic on delays in surgical procedures in Germany: a multi-center analysis of an administrative registry of 176,783 patients

**DOI:** 10.1186/s13037-022-00331-y

**Published:** 2022-06-28

**Authors:** Richard Hunger, Volker König, Rosi Stillger, René Mantke

**Affiliations:** 1 Department of General Surgery, University Hospital Brandenburg, Brandenburg Medical School Theodor Fontane, Hochstrasse 29, 14770 Brandenburg, Germany; 2 CLINOTEL Hospital Association gGmbH, Cologne, Germany; 3 Faculty of Health Sciences Brandenburg, Brandenburg Medical School Theodor Fontane, Brandenburg, Germany

**Keywords:** COVID-19, Elective surgery, Emergent surgery, General surgery, Health care research

## Abstract

**Background:**

While extensive data are available on the postponement of elective surgical procedures due to the COVID-19 pandemic for Germany, data on the impact on emergency procedures is limited.

**Methods:**

In this retrospective case–control study, anonymized case-related routine data of a Germany-wide voluntary hospital association (CLINOTEL association) of 66 hospitals was analyzed. Operation volumes, in-hospital mortality, and COVID-19 prevalence rates in digestive surgery procedure groups and selected single surgical procedures in the one-year periods before and after the outbreak of the COVID-19 pandemic were analyzed. The analysis was stratified by admitting department (direct admission or transfer to the general surgical department, i.e., primary or secondary surgical patients) and type of admission (elective/emergent).

**Results:**

The total number of primary and secondary surgical patients decreased by 22.7% and 11.7%, respectively. Among primary surgical patients more pronounced reductions were observed in elective (-25.6%) than emergency cases (-18.8%). Most affected procedures were thyroidectomies (-30.2%), operations on the anus (-24.2%), and closure of abdominal hernias (-23.9%; all *P*’s < 0.001). Declines were also observed in colorectal (-9.0%, *P* = 0.002), but not in rectal cancer surgery (-3.9%, n.s.). Mortality was slightly increased in primary (1.3 vs. 1.5%, *P* < 0.001), but not in secondary surgical cases. The one-year prevalence of COVID-19 in general surgical patients was low (0.6%), but a significant driver of mortality (OR = 9.63, *P* < 0.001).

**Conclusions:**

Compared to the previous year period, the number of patients in general and visceral surgery decreased by 22.7% in the first pandemic year. At the procedure level, a decrease of 14.8% was observed for elective procedures and 6.0% for emergency procedures. COVID-19 infections in general surgical patients are rare (0.6% prevalence), but associated with high mortality (21.8%).

**Trial registration:**

The present study does not meet the ICMJE definition of a clinical trial and was therefore not registered.

**Supplementary Information:**

The online version contains supplementary material available at 10.1186/s13037-022-00331-y.

## Introduction

Global outbreak of the COVID-19 pandemic was an unprecedented event in recent times with a unique impact on health systems worldwide [1–3]. One immediate measure to prepare healthcare systems for the impending patient volume and maintain intensive care bed capacity was the cancellation and postponement of elective surgical procedures [4, 5].

While general and nonspecific recommendations to protect patients, particularly cancer patients, from COVID-19 infection were available early [4, 5], detailed and specific recommendations for organizing, selecting, and performing surgical procedures, including ethical considerations, were not published until later in the COVID-19 pandemic [6]. Early recommendations and guidelines for patient selection regarding performance, deferral, or cancellation of a surgical procedure specifically addressed elective procedures [7, 8]. Early on, there was consensus in the recommendations that urgent and emergency surgeries should not be postponed [8, 9]. However, up to now, only single single-center data have been published on the impact on emergency procedures in general and visceral surgical care [10–12]. Furthermore, we are not aware of any studies that have investigated whether and how the patient pathway to surgery has changed. Specifically, whether the measures taken to manage the pandemic resulted in fewer patients being admitted directly to surgery (primary surgical cases) and, instead, increased numbers of patients through the detour of admission to other departments with subsequent transfer to surgery (secondary surgical cases).

The study aimed to assess the extent of decrease of surgical procedures, as well as the prevalence of COVID-19 infections among general surgery patients. Furthermore, we wanted to analyze the effect of the COVID-19 pandemic on in-hospital mortality and whether clinical pathways of patients to surgery changed due to the pandemic. Generally, we hypothesized a decrease in overall procedure volume in general surgery. For gastrointestinal surgery, the most important surgical area within general surgery, we predicted a mixed effect: while a reduction in volume is expected for elective procedures, such an effect was not predicted for emergency procedures. Regarding the patient pathways, we expect case number decreases mainly in primary surgical cases, while no changes or even slight case number increases are expected in secondary surgical cases, possibly due to compensation effects.

## Materials and methods

Data of the CLINOTEL hospital association, a voluntary association of 38 public and 28 non-profit, primarily medium-sized standard care hospitals distributed throughout Germany with approximately 31,000 beds, 1.3 million inpatients and 120,000 general surgical patients annually, was analyzed retrospectively. As data source routinely collected administrative data was used, that every German hospital is required to record by law ("Hospital Remuneration Act") and comprised of a range of information (e.g., gender, age, diagnoses, medical procedures) for each inpatient admission in a systematic and standardized way for reimbursement purposes. In 2019 hospitals of the CLINOTEL group performed 5.1% of all digestive surgery operations in Germany.

**Fig. 1:**
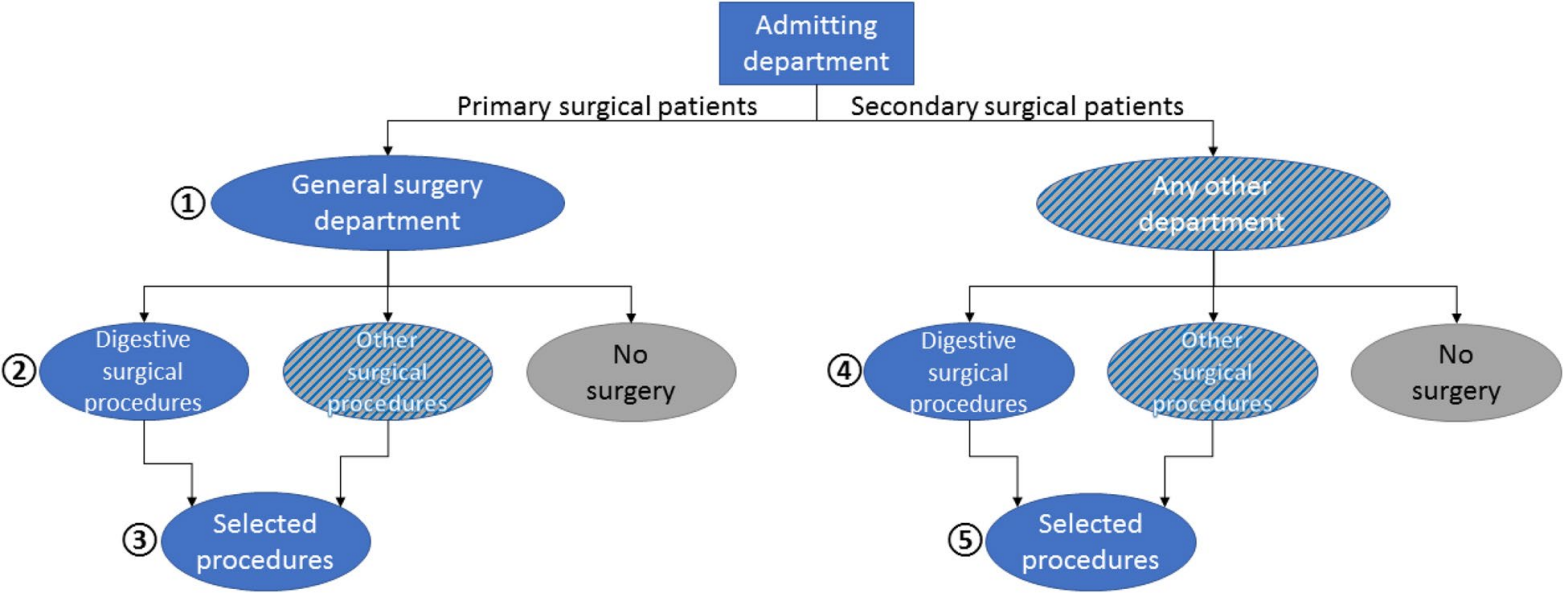
Graphical presentation of assessed patient groups. Note. Blue numbered ellipses indicate analyzed patient groups. Visceral surgery procedures include all procedures performed on the digestive tract. Selected procedures encompass thyroidectomy, appendectomy, cholecystectomy, as well as colon resections and rectum resection for cancer. For further details see text

Analyzed patient groups are summarized in Fig. 1. To assess different patient pathways, patients who underwent digestive surgery or single selected surgical procedures were identified and categorized as primary (direct admission to surgical department) or secondary surgical patients (admission to non-surgical departments). Digestive surgery procedures were identified according to subchapters of the German Classification System for Operations and Procedure Codes (OPS: chapters 5–42 to 5–54). Selected single procedures and their identifying OPS-codes were thyroidectomies (5–061 to 5–068), cholecystectomies as an independent procedure (5–511.0/1/2/x/y), appendectomies (5–470), and colon resection (5–455, 5–456, 5–484.0 and no rectum resection) or rectal resections (5–484.1 to 5–484.y, 5–485 and no concomitant colectomy 5–456) for colorectal cancer (ICD-10 main diagnosis C18, C19, C20, C21.8, D01.0, D01.2).

Two observation periods were defined in relation to the COVID-19 outbreak. Period 1 encompassed the period from 03/2019 to 02/2020 and period 2 from 03/2020 to 02/2021. Case assignment was made according to discharge date. Case numbers, procedure volumes and mortality rates were compared between study periods using chi-square tests and reported as Odds ratios (OR) with 95% confidence intervals (95%-CI). Analysis was stratified by admission type (elective/emergency). Comparison of mortality rates between observation periods and between patients with and without COVID-19 infection was performed using chi-square test. *P*-Values less than 0.05 were considered significant.

Analysis was performed with SPSS 26.0 (IBM Corp.) and R 4.0.5 (R Foundation for Statistical Computing). Reporting is in line with the STROBE guideline (Strengthening the Reporting of Observational studies in Epidemiology) [13]. By German law, ethical approval is not required for studies based on secondary data.

## Results

### Primary and secondary cases combined

**Fig. 2:**
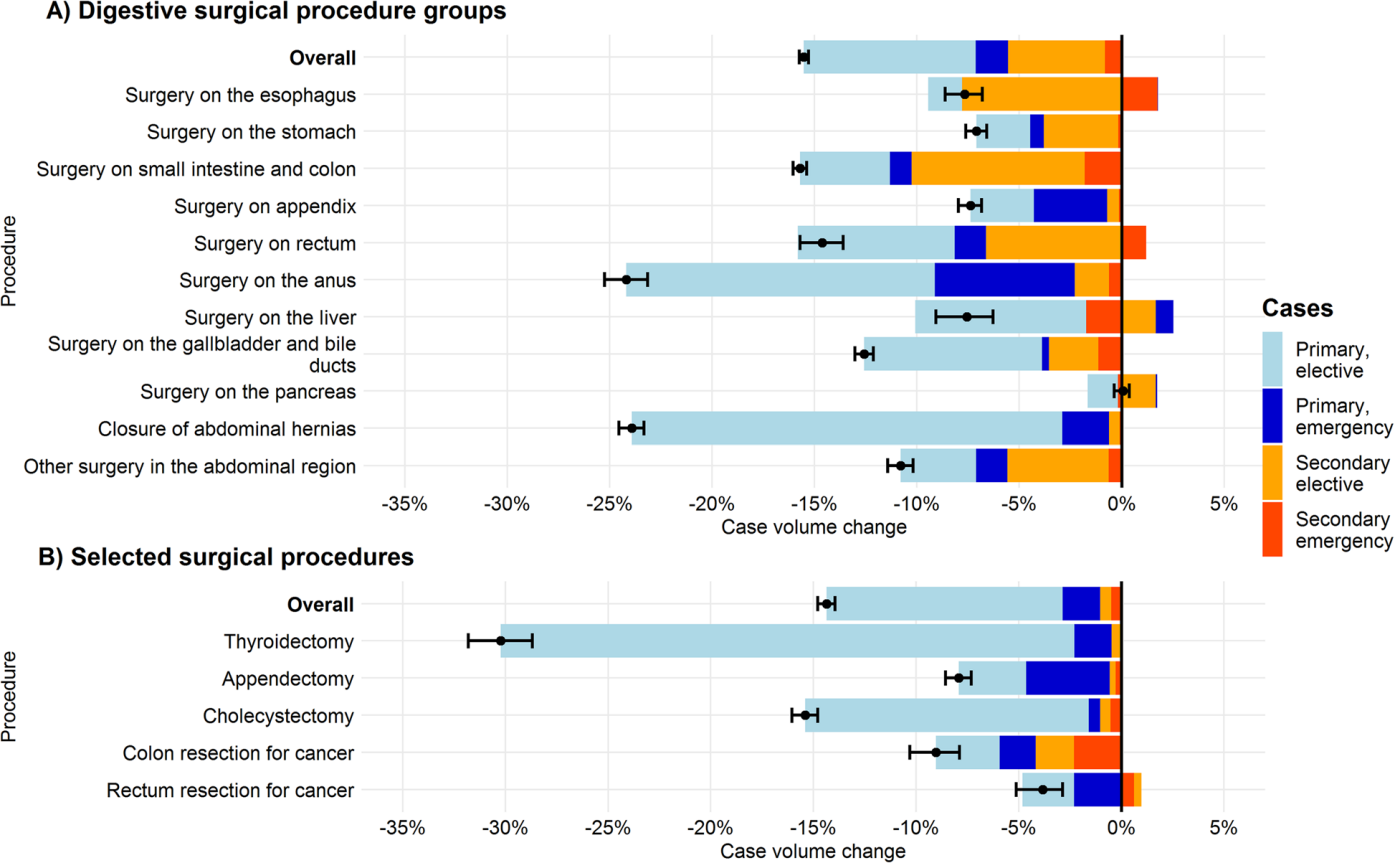
Case volume differences and distribution across patient groups. Note. Case volume differences between the two observation periods (03/2019–02/2020 vs. 03/2020–02/2021) stratified by primary/secondary patients (direct admission to surgical department and transferred from other departments, respectively) and admission type. Black dot and whiskers indicate overall case volume change and 95% confidence intervals. Stacked bar charts show the relative case volume change for the different patient groups. Bars right to the black line indicate an increased case volume. *N*_Period 1_ = 95,826. *N*_Period 2_ = 80,957

Overall case volume differences between the two observation periods, stratified by primary and secondary surgical cases and admission type, is shown in Fig. 2. Relative case volume differences and 95% confidence intervals are show, along with the contribution of the several patient groups to the case volume difference. A detailed overview of the results of the pooled primary and secondary surgical patient groups at the procedure level is provided in Table 1.

**Table 1:** Differences in procedure volumes and mortality between study periods (1-year pre/post COVID-19 outbreak in March 2020) and COVID-19 prevalence combined across *primary and secondary* surgical procedures

	Procedure volume (total)				Mortality (total)				Procedure volume (elective admissions)			
Group	Period 1 n	Period 2 n	Difference n (%)	*p*-value	Period 1 n (%)	Period 2 n (%)	OR [95% CI]	*p*-value	Period 1 n (%)	Period 2 n (%)	Difference n (%)	*p*-value
**Digestive surgical procedure groups (2 + 4); *N* = 176,783 patients**												
Surgery on the esophagus (5–42)	3,326	3,071	-255 (-7.7)	**.001**	168 (5.1)	186 (6.1)	1.21 [0.98, 1.50]	.089	1,938 (58.3)	1,642 (52.9)	-314 (-16.2)	** < .001**
Surgery on the stomach (5–43, 5–44)	9,596	8,916	-680 (-7.1)	** < .001**	662 (6.9)	621 (7.0)	1.01 [0.90, 1.13]	.882	5,517 (53.3)	4,516 (50.7)	-601 (-11.7)	** < .001**
Surgery on small intestine and colon (5–45, 5–46)	45,696	38,516	-7,180 (-15.7)	** < .001**	1,944 (4.3)	1,782 (4.6)	1.09 [1.02, 1.17]	**.009**	27,127 (59.4)	21,259 (55.2)	-5,868 (-21.6)	** < .001**
Surgery on appendix (5–47)	8,067	7,471	-596 (-7.4)	** < .001**	58 (0.7)	79 (1.1)	1.48 [1.05, 2.07]	**.030**	2,527 (31.3)	2,230 (29.8)	-297 (-11.8)	** < .001**
Surgery on rectum (5–48)	4,330	3,697	-633 (-14.6)	** < .001**	122 (2.8)	116 (3.1)	1.12 [0.86, 1.45]	.437	3.039 (70.2)	2,420 (65.5)	-619 (-20.4)	** < .001**
Surgery on the anus (5–49)	6,393	4,847	-1,546 (-24.2)	** < .001**	20 (0.3)	19 (0.4)	1.25 [0.67, 2.35]	.586	3,890 (60.8)	2,820 (58.2)	-1,070 (-27.5)	** < .001**
Surgery on the liver (5–50)	1,389	1,284	-105 (-7.6)	**.042**	97 (7.0)	86 (6.7)	0.96 [0.71, 1.29]	.829	955 (68.8)	862 (67.1)	-93 (-9.7)	**.029**
Surgery on the gallbladder and bile ducts (5–51)	21,112	18,458	-2,654 (-12.6)	** < .001**	538 (2.5)	504 (2.7)	1.07 [0.95, 1.21]	.272	11,928 (56.5)	9,586 (51.9)	-2,342 (-19.6)	** < .001**
Surgery on the pancreas (5–52)	1,559	1,560	1 (0.1)	.986	117 (7.5)	87 (5.6)	0.73 [0.55, 0.97]	**.035**	925 (59.3)	928 (59.5)	3 (0.3)	.944
Closure of abdominal hernias (5–53)	18,702	14,227	-4475 (-23.9)	** < .001**	144 (0.8)	148 (1.0)	1.35 [1.08, 1.71]	**.011**	15,052 (80.5)	11,024 (77.5)	-4,028 (-26.8)	** < .001**
Other surgery in the abdominal region (5–54)	9,568	8,535	-1,033 (-10.8)	** < .001**	927 (9.7)	838 (9.8)	1.01 [0.92, 1.12]	.788	5,375 (56.2)	4,548 (53.3)	-827 (-15.4)	** < .001**
**Selected surgical procedures (3 + 5); *N* = 48,684 patients**												
Thyroidectomy	3,315	2,313	-1,002 (-30.2)	** < .001**	6 (0.2)	8 (0.3)	1.91 [0.66, 5.52]	.342	3,057 (92.2)	2,116 (91.5)	-941 (-30.8)	** < .001**
Appendectomy	6,976	6,424	-552 (-7.9)	** < .001**	15 (0.2)	17 (0.3)	1.23 [0.61, 2.47]	**.006**	1,881 (27.0)	1,632 (25.4)	-249 (-13.2)	** < .001**
Cholecystectomy	12,712	10,754	-1,958 (-15.4)	** < .001**	143 (1.1)	160 (1.5)	1.33 [1.06, 1.67]	**.017**	7,444 (58.6)	5,624 (52.3)	-1,820 (-24.4)	** < .001**
Colon resection for cancer	2,161	1,966	-195 (-9.0)	**.002**	117 (5.4)	87 (4.4)	0.81 [0.61, 1.08]	.164	1,397 (64.6)	1,290 (65.6)	-107 (-7.7)	.309
Rectum resection for cancer	1,123	1,080	-43 (-3.8)	.360	30 (2.7)	34 (3.1)	1.18 [0.72, 1.95]	.509	872 (77.6)	848 (78.5)	-24 (-2.8)	.563

	Mortality (elective admissions)				Procedure volume (emergent admissions)				Mortality (emergent admissions)				COVID-19	
Group	Period 1 n (%)	Period 2 n (%)	OR [95% CI]	*p*-value	Period 1 n (%)	Period 2 n (%)	Difference n (%)	*p*-value	Period 1 n (%)	Period 2 n (%)	OR [95% CI]	*p*-value	Cases n (%)	Mortality n (%)
**Digestive surgical procedure groups (2 + 4); *N* = 176,783 patients**														
Surgery on the esophagus (5–42)	48 (2.5)	44 (2.7)	1.10 [0.72, 1.66]	.742	1,388 (41.7)	1,447 (47.1)	59 (4.3)	.268	120 (8.6)	142 (9.8)	1.15 [0.89, 1.48]	.313	30 (1.0)	6 (20.0)
Surgery on the stomach (5–43, 5–44)	209 (4.1)	178 (3.9)	0.96 [0.79, 1.18]	.761	4,479 (46.7)	4,400 (49.3)	-79 (-1.8)	.402	453 (10.1)	443 (10.1)	0.99 [0.87, 1.14]	.971	138 (1.5)	36 (26.1)
Surgery on small intestine and colon (5–45, 5–46)	668 (2.5)	541 (2.5)	1.03 [0.92, 1.16]	.585	18,569 (40.6)	17,248 (44.8)	-1,321 (-7.1)	** < .001**	1,276 (6.9)	1,241 (7.2)	1.05 [0.97, 1.14]	.240	264 (0.7)	77 (29.2)
Surgery on appendix (5–47)	25 (1.0)	27 (1.2)	1.23 [0.71, 2.12]	.553	5,540 (68.7)	5,241 (70.2)	-299 (-5.4)	**.004**	33 (0.6)	52 (1.0)	1.67 [1.08, 2.59]	.027	37 (0.5)	1 (2.7)
Surgery on rectum (5–48)	47 (1.5)	41 (1.7)	1.10 [0.72, 1.67]	.747	1,291 (29.8)	1,277 (34.5)	-14 (-1.1)	.782	75 (5.8)	75 (5.9)	1.01 [0.73, 1.41]	> .999	24 (0.6)	4 (16.7)
Surgery on the anus (5–49)	5 (0.1)	6 (0.2)	1.66 [0.51, 5.43]	.592	2,503 (39.2)	2,027 (41.8)	-476 (-19.0)	** < .001**	15 (0.6)	13 (0.6)	1.07 [0.51, 2.26]	> .999	14 (0.3)	1 (7.1)
Surgery on the liver (5–50)	56 (5.9)	48 (5.6)	0.95 [0.64, 1.41]	.865	434 (31.2)	422 (32.9)	-12 (-2.8)	.682	41 (9.4)	38 (9.0)	0.95 [0.60, 2.51]	.916	11 (0.9)	1 (9.1)
Surgery on the gallbladder and bile ducts (5–51)	219 (1.8)	183 (1.9)	1.04 [0.85, 1.27]	.732	9,184 (43.5)	8,872 (48.1)	-312 (-3.4)	**.020**	319 (3.5)	321 (3.6)	1.04 [0.89, 1.22]	.627	108 (0.6)	19 (17.6)
Surgery on the pancreas (5–52)	69 (7.5)	45 (4.8)	0.63 [0.43, 0.93]	**.025**	634 (40.7)	632 (40.5)	-2 (-0.3)	.955	48 (7.6)	42 (6.6)	0.87 [0.57, 1.34]	.595	13 (0.8)	2 (15.4)
Closure of abdominal hernias (5–53)	45 (0.3)	42 (0.4)	1.28 [0.84, 1.94]	.305	3,650 (19.5)	3,203 (22.5)	-447 (-12.2)	** < .001**	99 (2.7)	106 (3.3)	1.23 [0.93, 1.62	.169	35 (0.5)	11 (31.4)
Other surgery in the abdominal region (5–54)	332 (6.2)	268 (5.9)	0.95 [0.81, 1.12]	.583	4,193 (43.8)	3,987 (46.7)	-206 (-4.9)	**.023**	595 (14.2)	570 (14.3)	1.01 [0.89, 1.14]	.916	91 (1.1)	30 (33.0)
**Selected surgical procedures (3 + 5); *N* = 48,684 patients**														
Thyroidectomy	3 (0.1)	3 (0.1)	1.45 [0.29, 7.17]	.967	258 (7.8)	197 (8.5)	-61 (-23.6)	**.004**	3 (1.2)	5 (2.5)	2.21 [0.52, 9.38]	.456	5 (0.2)	0 (0.0)
Appendectomy	5 (0.3)	3 (0.2)	0.69 [0.16, 2.90]	.878	5,095 (73.0)	4,792 (74.6)	-303 (-5.9)	**.002**	10 (0.2)	14 (0.3)	1.49 [0.66, 3.36]	.445	28 (0.4)	0 (0.0)
Cholecystectomy	48 (0.6)	42 (0.7)	1.16 [0.77, 1.76]	.554	5,26841.4)	2,961 (27.5)	-2,307 (-43.8)	** < .001**	95 (1.8)	118 (4.0)	2.26 [1.72, 2.97]	** < .001**	45 (0.4)	11 (24.4)
Colon resection for cancer	56 (4.0)	49 (3.8)	0.95 [0.64, 1.40]	.856	764 (35.4)	676 (34.4)	-88 (-11.5)	**.020**	61 (8.0)	38 (5.6)	0.69 [0.45, 1.04]	.096	19 (1.0)	7 (36.8)
Rectum resection for cancer	17 (1.9)	20 (2.4)	1.21 [0.63, 2.34]	.676	251 (22.4)	232 (21.5)	-19 (-7.6)	.387	13 (5.2)	14 (6.0)	1.18 [0.54, 2.56]	.833	7 (0.6)	1 (14.3)

Proportion of elective and emergency procedures were reported in relation to procedure specific total volume. Mortality rates were calculated in relation to procedure volume and stratified by elective and emergency admissions. Results of testing for significant changes between time periods using chi-square test. Significant differences between the two observation periods are indicated in bold. *Sig.* Significance, *OR* Odds ratio. The numbers of (2) to (5) correspond to patient group definitions as in Fig. 1

**Fig. 3:**
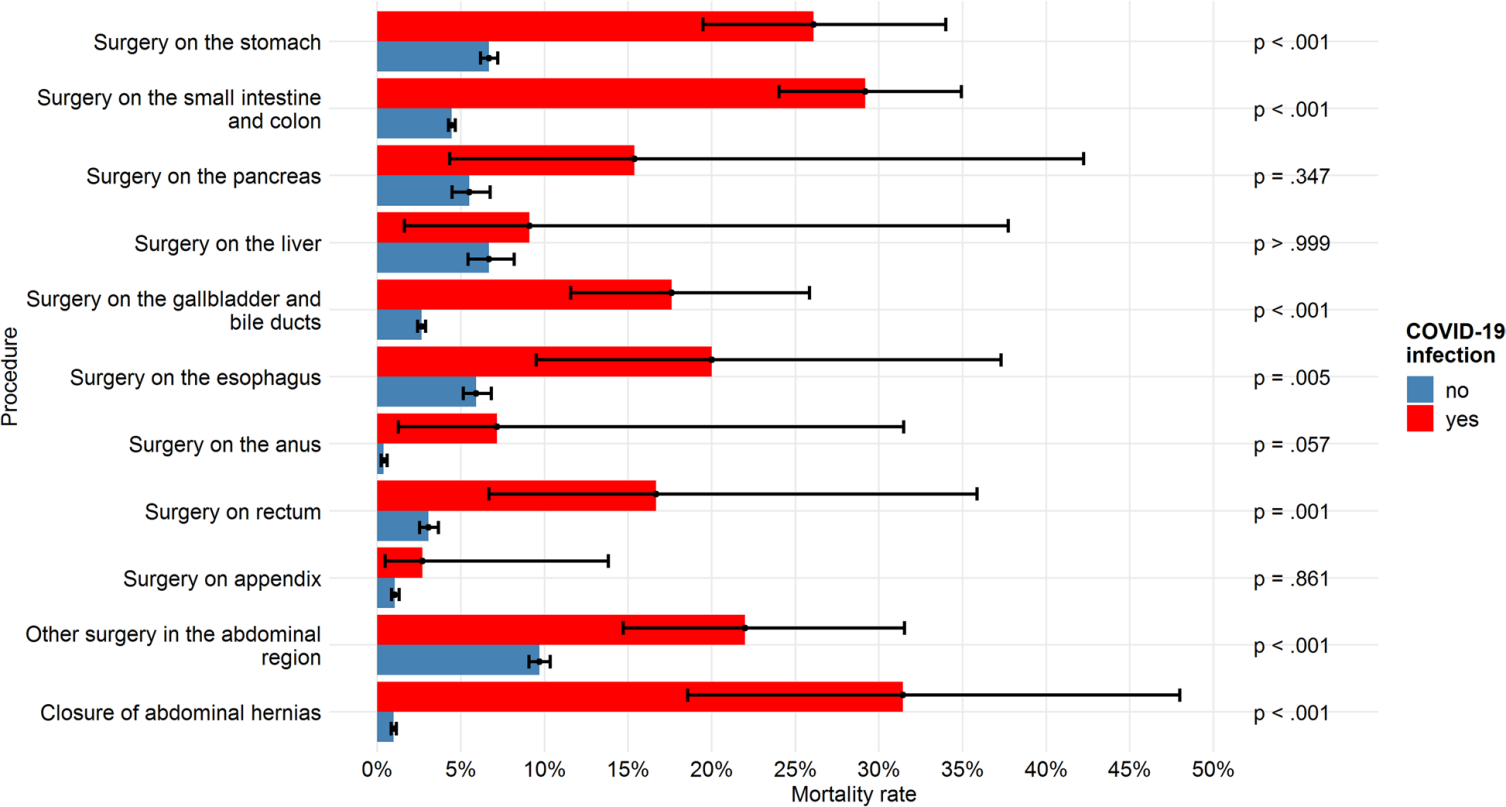
Mortality stratified by procedure group and COVID-19 infection status. Note. Mortality rates in the second observation period (03/2020–02/2021) across all digestive surgery patients stratified by procedure group and COVID-19 infection status. Black whiskers indicate 95% confidence intervals. *P* values indicate the results of chi-square tests comparing mortality rates between subgroups. Nno COVID-19 = 80,444. NCOVID-19 = 513

Across all digestive surgical procedure groups, mortality in the first period was 2.7% (*n* = 2,544) and in the second period 2.9% (*n* = 2.379), indicating a significant increase in mortality (OR = 1.11, 95%-CI: [1.05; 1.17], *P* < 0.001). A COVID-19 infection was present in 0.6% patients (513 of 80,957). Cross classification of COVID-19 status and mortality of digestive surgery patients revealed significantly increased mortality among COVID-19 positive patients (21.8%, *n* = 112) compared to COVID-19 negative (2.8%, *n* = 2,267) with an Odds Ratio of 9.6 (95%-CI: 7.78 to 11.93, *P* < 0.001). Mortality rates for the various digestive surgical procedures stratified by COVID-19 infection status are shown in Fig. 3. Only 4 groups of procedures did not show significant mortality differences (surgery on the appendix, anus, liver, and pancreas).

**Fig. 4:**
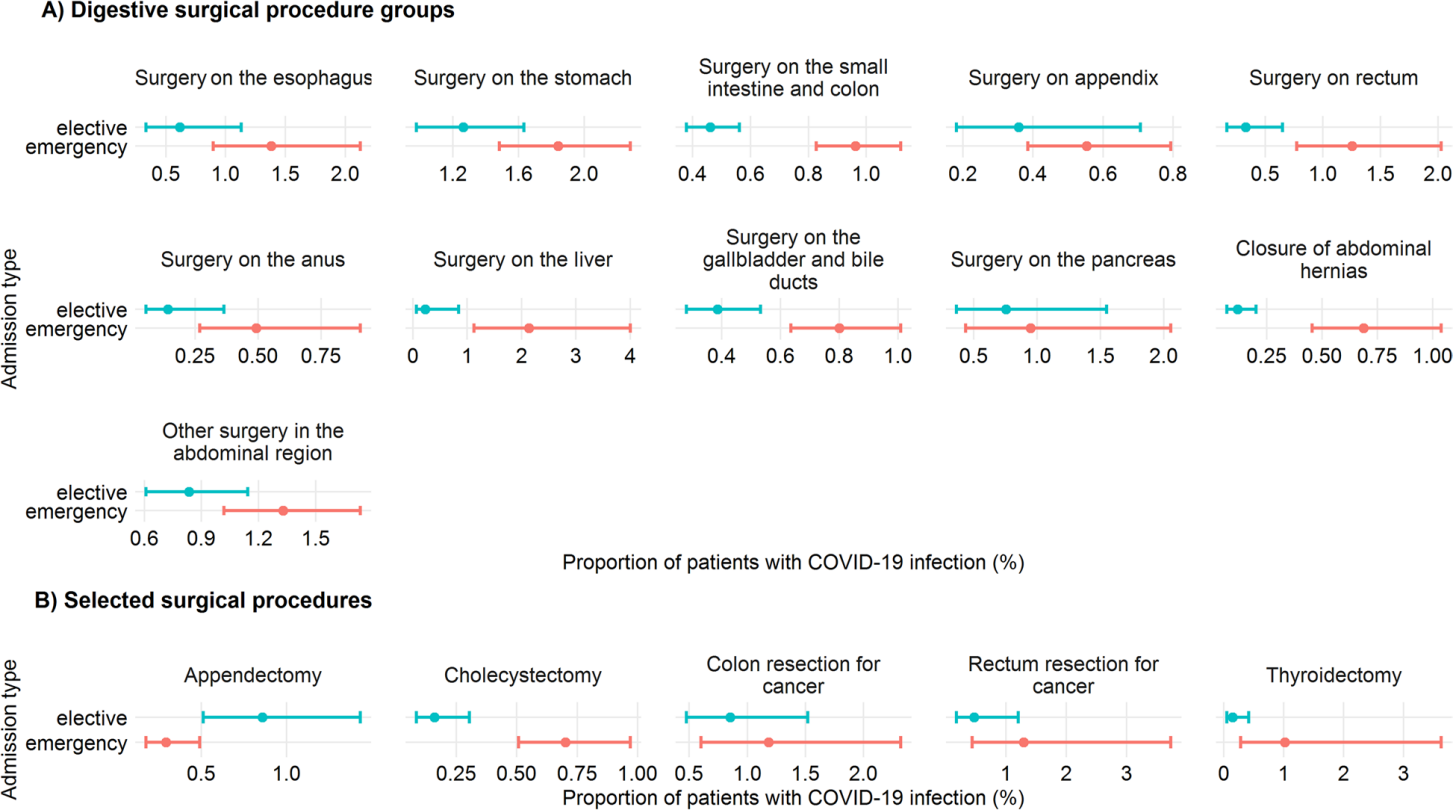
COVID-19 prevalence stratified by procedure and admission type. Note*.* Proportion of patients with concomitant COVID-19 infection (point estimate and 95% confidence interval) in digestive surgery (**A**) and selected procedures (**B**) stratified by admission type. Combined analysis of primary and secondary surgical cases. Varying N, see Table 1 for further details

COVID-19 prevalence, stratified by elective and emergency admission is given in Fig. 4. Except for appendectomies, the proportion of patients with COVID-19 diagnosis was consistently lower in elective than in emergency admissions. While 8860 individuals (4.2%) received intensive care in the first observation period, the number increased to 9455 (5.2%) in the second period. Of these 5.2%, 1,641 cases had COVID-19 coding (17.4%).

### Primary surgical cases

The number of primary surgical patients decreased from 163,758 in the first to 126,561 in the second period by -22.7%. These reductions could be observed for elective (-25.6%) and emergency cases (-18.8%). Digestive surgery procedures were affected by a less pronounced decline in primary case volume, from 50,634 to 41,074 cases (-18.9%). A decrease of 3,495 cases (-17.1%) was observed in selected surgical procedures.

In elective admissions, a significant volume decline was noted for 10 of the 11 digestive procedure groups (except *operations on the pancreas*). For emergent admissions the decline was less pronounced and only significant in 6 of 11 digestive surgical procedure groups (Additional Table S1).

Of the selected surgical procedures, thyroidectomies and cholecystectomies had the strongest decline in elective procedure volume. Remarkably, even in the case of emergency appendectomies, a significant decrease in the number of procedures was recorded (-7.2%, *p* = 0.001), while emergency cholecystectomy volume was unchanged (-2.5%, ns). A significant procedure volume reduction was observed in colon cancer (-7.7%, *p* = 0.04), but not in rectal cancer (-5.8%, ns).

Overall case burden in the hospital group of COVID-19 cases in the observation period was 17,484 (9.6% of all inpatient admissions). COVID-19 among all primary surgical patients in the second analysis period was observed in 741 cases (0.6%). Stratified by admission type, COVID-19 infections were observed more frequently in emergent than in elective admissions (0.9% and 0.3%, respectively).

In primary surgical patients undergoing digestive tract surgery, 136 cases (0.3%) were diagnosed with COVID-19. Again, a higher proportion was found in emergency than in regular cases (0.6% and 0.2%, respectively). Regarding individual digestive procedure groups, COVID-19 infection rates ranges from 0.2% to 1.8%. In selected surgical procedures, a total of 49 patients (0.3%) had COVID-19 infection ranging from 0 to 1% for specific procedures (Additional Table S1).

Across all primary surgical patients, mortality was significantly increased in the second period (1.3% versus 1.5%, OR = 1.17, *P* < 0.001). Overall mortality rates in both, elective and emergency surgery, increased slightly (0.7% vs. 0.8%, OR = 1.12, *P* = 0.042, and 2% vs. 2.3%, OR = 1.15, *P* < 0.001, respectively). With the exception of *surgery on the liver*-procedures, no significant mortality differences were observed in the digestive surgical procedure groups. In the selected surgical procedures, only in *rectum resections for cancer* a significantly increased mortality rate was observed (Additional Table S1).

### Secondary surgical cases

In secondary surgical patients less pronounced case number declines than for primary surgical patients were observed. The number of digestive surgery patients decreased from 45,192 to 39,883 cases (-11.7%, *P* < 0,001). Even smaller case number declines were observed in selected surgical procedures (from 5,799 to 5,528 cases, -4.7%, *P* = 0,011).

The results of the analysis on procedure level for the group of secondary surgical patients are summarized in Additional Table S2. Significant decreases in procedure volume were noted in 8 of the 11 digestive surgical procedure groups (exceptions: *surgery on the appendix, liver, and pancreas*). Except for two procedure groups, there was also a significant decrease for all other elective procedures. The decline in emergency admissions was less pronounced and only significant in 2 of 11 digestive surgical procedure groups (*surgery on the small intestine and colon and surgery on the gallbladder*).

Regarding selected surgical procedures, there was only a significant reduction in the number of *resections in colon cancer* (-11.2%, *P* = 0.021; Table S2). Stratified by admission type, no significant changes were observed in elective or emergent cases.

In secondary surgical patients undergoing digestive tract surgery, 377 cases (0.9%) were diagnosed with COVID-19. This proportion was three times higher than in primary digestive surgery patients. Only in *surgery on the esophagus* and *colon resection for cancer* a lower proportion was observed. As in primary surgical patients, higher COVID-19 rates were found in emergency than in elective cases (0.6% and 0.2%, respectively). In selected surgical procedures, COVID-19 infections were observed in 1% of patients (*n* = 55) with values ranging from 0.9% to 3.0% in individual procedures (Additional Table S2).

Overall mortality in all secondary surgical cases remained unchanged (1.6% vs. 1.5%, *P* = 0.253). In digestive surgery only *surgery on small intestine and colon* had an increased mortality rate (4.1% vs. 4.5%, *P* = 0.011). In selected surgical procedures, the overall mortality rate in rectum resection for cancer was reduced (10.3% vs. 4.4%, *P* = 0.037), while in emergent cholecystectomies mortality increased (2.6% vs. 3.8%, *P* = 0.022, Additional Table S2).

## Discussion

The present study examined the effect of the COVID-19 pandemic on case volume and in-hospital mortality, as well as the prevalence of COVID-19 in general surgery patients in a multi-institutional setting of 66 general hospitals across Germany, with special regard to emergency procedures. In march 13^th^ 2020 ministry of health issued a request to all German hospitals to postpone elective surgical procedures without further guidelines [14]. Decision and patient selection were left to the discretion of the treating surgeon, in contrast to other countries, where guidance on selecting patients for surgery was provided [7]. How the guidelines were actually implemented, i.e. which operations were postponed, to what extent emergency procedures were affected, and whether and how patient pathways changed, was analyzed in the present study for Germany.

The analysis showed a decrease of 22.7% for the total number of patients in general and visceral surgery was observed, a decrease in the order of magnitude as in the international modelling study [16] and as observed by studies on individual procedures [10–12, 18]. However, this number includes all inpatients admitted to general surgery departments, regardless whether surgery was actually performed or not, and thus tends to be an inflated estimate. More valid figures emerge when considering digestive surgery patients. This group of patients showed substantially lower decreases in the number of cases (-15.5%) and procedures (-14.8%), and are thus considerably below the modeled figures. An extrapolation of the observed cancellation rates across Germany would therefore yield a backlog of approximately 376,000 digestive operations.

A methodologic sophisticated study predicted the cessation of 75,730 elective operations in Germany weekly during the first wave of the pandemic, based on experts’ expectations about surgical cancellation rates [16]. Specifically, for cancer surgery the model estimated a cancellation rate of 24.0% (range: 17.2%—42.3%) for Germany. Thus, the backlog of operations in general and visceral surgery is less than initially expected [15]. For the modeling, however, various assumptions had to be postulated (e.g. duration of lockdown, surgical case-mix) and estimates based on expected projections of senior surgeons. Deviations from the modeled figures could be explained by an immediate catch-up effect. The modeled predictions are based on the 12-week peak of infection during the first wave, when cancellation rates were highest. However, the observation period of the present study covered the entire first year of the pandemic and since COVID infection rates showed a strong seasonal effect, with low incidence in the summer months, it is likely that postponed surgeries were rescheduled immediately. This can possibly be explained by the fact that the predictions and observations in the studies reported were based on the lockdown phases, in which the decline in the number of cases was particularly pronounced. Immediate catch-up effects, as they were observed for some procedures in our study, were not taken into account in other studies.

As hypothesized, the decrease in procedure volume was less pronounced in secondary than in primary surgery patients. The only exceptions were observed in surgery on small intestine/colon and colon resection for cancer, where the decrease in the number of cases was more pronounced than in primary surgical patients. This finding suggests that the surgical care of patients in the sense of co-treatment of patients admitted to other departments was generally less affected. This indicates an alternative care pathway through which patients received surgical treatment.

So far, there are no extensive multi-center analyses for general surgery on the impact on the case volume that differentiated between elective and emergency cases. As hypothesized, case volume decline was more pronounced in elective than in emergency surgery. Majority of postponed procedures were observed in primary surgical elective cases. Unexpectedly, there were also significant and, in some cases, even substantial decreases in the number of procedures for patients admitted as emergencies (e.g. *surgery on the anus*, -19.0%). Although less accentuated than in elective procedures, this decline is unexpected, since only scheduled operations should be postponed and at no time there were recommendations to postpone or cancel emergent operations [8, 9]. Two possible explanations can be considered for this effect. First, the utilization behavior of patients could have changed in such a way that they tended to avoid hospitalization, especially during periods of high COVID-19 infection rates. On the other hand, conservative treatment methods may have been used more frequently in order to avoid necessary stays in intensive care units as a result of surgical complications.

Interestingly, for some procedures we observed a compensating effect of increased case volumes in secondary surgical cases and a shift to more emergency procedures. This finding is supported by case volume changes in single procedures, with the largest decline in case numbers for thyroidectomies, as an almost entirely electively performed procedure. There the number of cases dropped by a third, compared to the same period of the previous year. Second most affected were cholecystectomies, with a case mix of elective and emergent indications. Less pronounced were the case reduction in appendectomies and colorectal cancer surgery.

Data from two single hospitals on appendectomy and cholecystectomy case volumes showed a decrease of about 50% during the first pandemic wave compared to the same period of the previous year (calendar week 12—20) [11]. Another study on appendectomies, that additionally differentiated according to the indication, reported this decrease specifically for simple and non-complex appendicitis, while not for complex acute appendicitis [10]. A reanalysis of the published data showed a decrease in procedures of 17.1% compared to the same period in the previous year (calendar week 12—17).

The descriptive analysis showed an increase in mortality for most of the procedures and groups of procedures, but only a few actually showed significant differences. A COVID-19 infection showed a highly significant association with increased mortality rates (OR = 9.6, 95% CI: 7.78 to 11.93). The strength of the association is comparable to that observed in an Italian single-center (OR = 9.5) [17] and a two-center study (RR_adjusted_ = 9.29) from New York [18]. However, the increased mortality is probably not only due to the additional risk factor of a COVID-19 infection. Rather, the surgical case mix has shifted towards more complicated interventions and more emergent procedures, which are associated with increased mortality [10, 11]. Future studies had to disentangle the effect of a changed surgical case-mix and increased mortality because of preoperative COVID-19 infection [19].

Generally, COVID-19 prevalence was low in the study group. Of the patients directly admitted to general surgical department, only a very small percentage were COVID-19 positive. This can be explained by the fact that all patients underwent a SARS-CoV2 antigen rapid test before hospitalization. In case of a positive rapid test result, an additional PCR test was performed and patients were isolated in the ward until the test result was obtained. An above average proportion of infections was observed in patients treated in the upper gastrointestinal tract (esophagus, stomach). This probably reflects the clinical component of the increased prevalence of gastrointestinal symptoms in COVID-19 infection [20].

The analysis based on administrative data primarily collected for remuneration issues and has the usual limitations of such data, mainly incomplete clinical data and non-stringent definition of mortality (in-hospital vs. 30d) [21]. Furthermore, it must be noted that the hospital network mainly comprises medium-sized hospitals. Therefore, an underestimation of the observed effects cannot be ruled out, since complicated COVID-19 cases in particular tended to be treated more frequently at university hospitals. Possible differences between university hospitals and other standard care hospitals are indicated by single-center data, which described a significant decline in the number of cases for cholecystectomies only at university hospitals [11]. With 23.8% more intensive care cases in the second observation period, a considerable increase in intensive care cases could be observed in the hospital sample examined. This increase is in the range of the value observed by a Germany-wide model for intensive care units [22], which indicates that the hospitals of the sample are comparable in this value with the totality of German hospitals.

Another restriction arises in the estimate of the backlog of operations, as we have only calculated the decrease in the number of operations compared to the previous year’s values, while the number of inpatient operations generally increase by about 1.8% each year [23]. Therefore, the numbers may underestimate the real backlog of operations. Finally, the specificity of the situation in Germany during the pandemic must be pointed out, such as low COVID-19 case numbers in international comparison or no uniform national response strategy. These limitations should be considered when generalizing the results to other countries and regions.

It has been shown that the impact of a pre-operative COVID-19 diagnosis on 30-day post-operative mortality diminishes over time with a practically equal risk of mortality with undiagnosed patients after 7 weeks [19]. The dataset provided no opportunity to control for the time-differences between COVID-19 diagnosis and date of operation. Furthermore, because of rare mortality events in combination with homogeneous patient groups, risk-adjusted modelling was not feasible.

## Conclusion

In summary, it can be concluded that the backlog of surgeries in general and visceral surgery is less than early predictions suggested. Primary surgical patients were more affected, while the care of secondary surgical patients, i.e. those who needed general surgical care during a hospital stay in other departments, was largely assured. The proportion of COVID-19 in all general surgical cases was generally low (0.6%), but a significant driver of mortality (OR = 9.63, *P* < 0,001).

## Supplementary Information

**Additional file 1: Table S1.** Differences in procedure volumes and mortality between the two observation periods (1-year pre/post COVID-19 outbreak in March 2020) and COVID-19 prevalence in *primary* surgical procedures.

**Additional file 2: Table S2.** Differences in procedure volumes and mortality between the two observation periods (1-year pre/post COVID-19 outbreak in March 2020) and COVID-19 prevalence in *secondary* surgical procedures.

## Data Availability

The data that support the findings of this study are available from CLINOTEL Group gGmbH but restrictions apply to the availability of these data, which were used under license for the current study, and so are not publicly available. Data are however available from the authors upon reasonable request and with permission of the CLINOTEL Group gGmbH.
