# Supplementary material for: Impact of the COVID-19 pandemic on delays in surgical procedures in Germany: a multi-center analysis of an administrative registry of 176,783 patients
Source: Patient Saf Surg. 2022 Jun 28;16:22. doi: 10.1186/s13037-022-00331-y (PMC9238103; doi:10.1186/s13037-022-00331-y)
Supplement: Supplementary file 2 — Additional file 2: Table S2. Differences in procedure volumes and mortality between the two observation periods (1-year pre/post COVID-19 outbreak in March 2020) and COVID-19 prevalence in secondary surgical procedures. [file 13037_2022_331_MOESM2_ESM.docx]

Table S2. Differences in procedure volumes and mortality between the two observation periods (1-year pre/post COVID-19 outbreak in March 2020) and COVID-19 prevalence in ***secondary* surgical procedures**

|  | | Procedure volume (total) | | | | Mortality (total) | | | | Procedure volume (elective admissions) | | | | Mortality (elective admissions) | | | | | Procedure volume (emergent admissions) | | | | Mortality (emergent admissions) | | | | | COVID-19 | | |  |
| --- | --- | --- | --- | --- | --- | --- | --- | --- | --- | --- | --- | --- | --- | --- | --- | --- | --- | --- | --- | --- | --- | --- | --- | --- | --- | --- | --- | --- | --- | --- | --- |
|  | Group | Period 1 n | Period 2 n | Difference n (%) | p-value | Period 1 n (%) | Period 2 n (%) | OR [95% CI] | p-value | Period 1 n (%) | Period 2 n (%) | Difference n (%) | p-value | Period 1 n (%) | Period 2 n (%) | OR [95% CI] | p-value | Period 1 n (%) | | Period 2 n (%) | Difference n (%) | p-value | Period 1 n (%) | Period 2 n (%) | OR [95% CI] | p-value | Cases n (%) | | Mortality n (%) |  |  |
| **Digestive surgical procedure groups (4); N = 85,075 patients** | | | | | | | | | | | | | | | | | | | | | | | | | | | | | | | |
|  | Surgery on the esophagus (5-42) | 2,990 | 2,789 | -201 (-6.7) | **.008** | 154 (5.2) | 166 (6.0) | 1.17 [0.93, 1.46] | .203 | 1,678 (56.1) | 1,419 (50.9) | -259 (-15.4) | **< .001** | 41 (2.4) | 34 (2.4) | 0.98 [0.62, 1.55] | > .999 | 1,312 (43.9) | | 1,370 (49.1) | 58 (4.4) | .263 | 113 (8.6) | 132 (9.6) | 1.13 [0.87, 1.47] | .394 | 25 (0.9) | | 4 (16.0) |  |  |
|  | Surgery on the stomach (5-43, 5-44) | 6,567 | 6,202 | -365 (-5.6) | **< .001** | 533 (8.1) | 523 (8.4) | 1.04 [0.92, 1.18] | .537 | 2,704 (41.2) | 2,355 (38.0) | -349 (-12.9) | **< .001** | 147 (5.4) | 130 (5.5) | 1.01 [0.80, 1.30] | .945 | 3,863 (58.5) | | 3,847 (62.0) | -16 (-0.4) | .572 | 386 (10.0) | 393 (10.2) | 1.03 [0.88, 1.89] | .774 | 126 (2.0) | | 31 (24.6) |  |  |
|  | Surgery on small intestine and colon (5-45, 5-46) | 29,515 | 24,826 | -4,689 (-15.9) | **< .001** | 1,208 (4.1) | 1,127 (4.5) | 1.11 [1.03, 1.21] | **.011** | 16,688 (56.5) | 12,825 (51.7) | -3,863 (-23.1) | **< .001** | 381 (2.3) | 283 (2.2) | 0.97 [0.83, 1.23] | .690 | 12,827 (43.5) | | 11,992 (48.3) | -835 (-6.5) | **< .001** | 827 (6.4) | 844 (7.0) | 1.09 [1.00, 1.21] | .067 | 182 (0.7) | | 55 (30.2) |  |  |
|  | Surgery on appendix (5-47) | 2,045 | 1,987 | -58 (-2.8) | .361 | 31 (1.5) | 42 (2.1) | 1.40 [0.88, 2.24] | .192 | 682 (33.3) | 635 (32.0) | -47 (-6.9) | .195 | 12 (1.8) | 10 (1.6) | 0.89 [0.38, 2.08] | .963 | 1,363 (66.7) | | 1,352 (68.0) | -11 (-0.8) | .833 | 19 (1.4) | 32 (2.4) | 1.71 [0.97, 3.04] | .084 | 19 (1.0) | | 1 (5.3) |  |  |
|  | Surgery on rectum (5-48) | 1,985 | 1,750 | -235 (-11.8) | **< .001** | 83 (4.2) | 68 (3.9) | 0.93 [0.67, 1.29] | .708 | 1,212 (61.1) | 925 (52.9) | -287 (-23.7) | **< .001** | 28 (2.3) | 18 (1.9) | 0.84 [0.46, 1.53] | .671 | 773 (38.9) | | 825 (47.1) | 52 (6.7) | .193 | 55 (7.1) | 50 (6.1) | 0.84 [0.57, 1.25] | .454 | 14 (0.8) | | 3 (21.4) |  |  |
|  | Surgery on the anus (5-49) | 775 | 628 | -147 (-19.0) | **< .001** | 13 (1.7) | 7 (1.1) | 0.66 [0.26, 1.67] | .511 | 361 (46.6) | 254 (40.4) | -107 (-29.6) | **< .001** | 4 (1.1) | 1 (0.4) | 0.35 [0.04, 3.17] | .606 | 414 (53.4) | | 374 (59.6) | -40 (-9.7) | .154 | 9 (2.2) | 6 (1.6) | 0.73 [0.26, 2.08] | .746 | 6 (1.0) | | 1 (16.7) |  |  |
|  | Surgery on the liver (5-50) | 477 | 476 | -1 (-0.2) | .974 | 44 (9.2) | 57 (12.0) | 1.34 [0.88, 2.03] | .203 | 207 (43.4) | 230 (48.3) | 23 (11.1) | .271 | 15 (7.2) | 22 (9.6) | 1.35 [0.68, 2.69] | .486 | 270 (56.6) | | 246 (51.7) | -24 (-8.9) | .291 | 29 (10.7) | 35 (14.2) | 1.38 [0.81, 2.33] | .286 | 7 (1.5) | | 1 (14.3) |  |  |
|  | Surgery on the gallbladder and bile ducts (5-51) | 10,308 | 9,557 | -751 (-7.3) | **< .001** | 384 (3.7) | 381 (4.0) | 1.07 [0.93, 1.24] | .358 | 4,257 (41.3) | 3,747 (39.2) | -510 (-12.0) | **< .001** | 135 (3.2) | 107 (2.9) | 0.90 [0.69, 1.16] | .449 | 6,051 (58.7) | | 5,810 (60.8) | -241 (-4.0) | **.027** | 249 (4.1) | 274 (4.7) | 1.15 [0.97, 1.37] | .121 | 81 (0.8) | | 15 (18.5) |  |  |
|  | Surgery on the pancreas (5-52) | 1,095 | 1,118 | 23 (2.1) | .625 | 65 (5.9) | 54 (4.8) | 0.80 [0.55, 1.17] | .290 | 543 (49.6) | 569 (50.9) | 26 (4.8) | .436 | 30 (5.5) | 17 (3.0) | 0.53 [0.29, 0.97] | .051 | 552 (50.4) | | 549 (49.1) | -3 (-0.5) | .928 | 35 (6.3) | 37 (6.7) | 1.07 [0.66, 1.72] | .884 | 12 (1.1) | | 2 (16.7) |  |  |
|  | Closure of abdominal hernias (5-53) | 1,641 | 1,526 | -115 (-7.0) | **.041** | 64 (3.9) | 74 (4.8) | 1.26 [0.89, 1.77] | .222 | 929 (56.6) | 832 (54.5) | -97 (-10.4) | **.021** | 15 (1.6) | 18 (2.2) | 1.35 [0.67, 2.69] | .502 | 712 (43.4) | | 694 (45.5) | -18 (-2.5) | .631 | 49 (6.9) | 56 (8.1) | 1.19 [0.80, 1.77] | .456 | 12 (0.8) | | 4 (33.3) |  |  |
|  | Other surgery in the abdominal region (5-54) | 5,139 | 4,604 | -535 (-10.4) | **< .001** | 568 (11.1) | 495 (10.8) | 0.97 [0.85, 1.10] | .657 | 2,855 (55.6) | 2,381 (51.7) | -474 (-16.6) | **< .001** | 183 (6.4) | 132 (5.5) | 0.86 [0.68, 1.08] | .210 | 2,284 (44.4) | | 2,223 (48.3) | -61 (-2.7) | .364 | 385 (16.9) | 363 (16.3) | 0.96 [0.82, 1.13] | .663 | 57 (1.2) | | 17 (29.8) |  |  |
| **Selected surgical procedures (5); N = 11,327 patients** | | | | | | | | | | | | | | | | | | | | | | | | | | | | | | | |
|  | Thyroidectomy | 148 | 132 | -16 (-10.8) | .339 | 5 (3.4) | 6 (4.5) | 1.36 [0.41, 4.57] | .846 | 102 (68.9) | 87 (65.9) | -15 (-14.7) | .275 | 2 (2.0) | 2 (2.3) | 1.18 [0.16, 8.53] | > .999 | 46 (31.1) | | 45 (34.1) | -1 (-2.2) | .917 | 3 (6.5) | 4 (8.9) | 1.40 [0.29, 6.63] | .976 | 4 (3.0) | | 0 (0.0) |  |  |
|  | Appendectomy | 1,526 | 1,486 | -40 (-2.6) | .466 | 8 (0.5) | 7 (0.5) | 0.90 [0.32, 2.48] | > .999 | 361 (23.7) | 340 (22.9) | -21 (-5.8) | .428 | 3 (0.8) | 1 (0.3) | 0.35 [0.04, 3.40] | .659 | 1,165 (76.3) | | 1,146 (77.1) | -19 (-1.6) | .693 | 5 (0.4) | 6 (0.5) | 1.22 [0.37, 4.01] | .978 | 14 (0.9) | | 0 (0.0) |  |  |
|  | Cholecystectomy | 3,158 | 3,026 | -132 (-4.2) | .093 | 94 (3.0) | 111 (3.7) | 1.24 [0.94, 1.64] | .148 | 680 (21.5) | 616 (20.4) | -64 (-9.4) | .075 | 29 (4.3) | 19 (3.1) | 0.71 [0.40, 1.29] | .329 | 2,478 (78.5) | | 2,410 (79.6) | -68 (-2.7) | .331 | 65 (2.6) | 92 (3.8) | 1.47 [1.07, 2.03] | **.022** | 27 (0.9) | | 9 (33.3) |  |  |
|  | Colon resection for cancer | 800 | 710 | -90 (-11.2) | **.021** | 60 (7.5) | 42 (5.9) | 0.78 [0.52, 1.17] | .262 | 375 (46.9) | 335 (47.2) | -40 (-10.7) | .133 | 23 (6.1) | 20 (6.0) | 0.97 [0.52, 1.80] | > .999 | 425 (53.1) | | 375 (52.8) | -50 (-11.8) | .077 | 37 (8.7) | 22 (5.9) | 0.65 [0.38, 1.13] | .162 | 7 (1.0) | | 4 (57.1) |  |  |
|  | Rectum resection for cancer | 195 | 206 | 11 (5.6) | .583 | 20 (10.3) | 9 (4.4) | 0.40 [0.18, 0.90] | **.037** | 100 (51.3) | 104 (50.5) | 4 (4.0) | .779 | 11 (11.0) | 3 (2.9) | 0.24 [0.06, 0.89] | **.044** | 95 (48.7) | | 102 (49.5) | 7 (7.4) | .618 | 9 (9.5) | 6 (5.9) | 0.60 [0.20, 1.75] | .496 | 3 (1.5) | | 1 (33.3) |  |  |

Note. Secondary surgical patients were all patients relocated from other departments to general surgical department. Proportion of elective and emergency procedures were reported in relation to procedure specific total volume. Mortality rates were calculated in relation to procedure volume and stratified by elective and emergency admissions. Results of testing for significant changes between time periods using chi-square test. Significant differences between the two observation periods are indicated in bold. Sig., Significance. OR, Odds ratio. (4) and (5) correspond to patient group definitions as in Figure 1
